# Supplementary material for: Validation of a Cantonese Version of the Amsterdam-Nijmegen Everyday Language Test (CANELT): A functional approach
Source: PLoS One. 2024 May 24;19(5):e0303810. doi: 10.1371/journal.pone.0303810 (PMC11125462; doi:10.1371/journal.pone.0303810)
Supplement: S1 Appendix — (DOCX) [file pone.0303810.s001.docx]

**Appendix A**

**Modified CANELT scenarios (translated in English) and reasons for modifications**

1. You are at the butcher’s, and this (*a hundred-dollar banknote*) is on the floor. What do you say to the butcher?

(*Original scenario in the English version: You are at the butcher’s, and this (a glove) is on the floor. What do you say? Reason for modification: It is unusual to see a glove on the floor at a local butcher’s shop.)

1. Your neighbor’s dog is barking all day long. Your patience is running out. You are calling to the management office of the building and what do you say?

(*Original version in English: Your neighbor’s dog is barking all day long. Your patience is running out. You want to talk to him about it. What do you say? Reason for modification: People in Hong Kong usually live in buildings and they usually make a complaint to the management office instead of talking to the dog owner directly.)

1. Now you are the salesperson. I want to buy one of these [*showing two radio receivers]* and I ask “Which one is better”? You have to recommend me something. What do you say?

(*Original version in English: Now you are the salesperson. I want to buy one of these things (corkscrews). You have to recommend me something. - “Which one is better”? Reason for modification: Corkscrew is not a common household item among Hong Kong families.)

1. You are in the cinema and want to watch a movie. What do you say when you are at the ticket office?

(*Original version in English: You are at the train station. You need to go to [place]. You are near the ticket office. What do you say? Reason for modification: People in Hong Kong usually tap their octopus card/credit card for train tickets.)

1. You saw a cat strolling at your back stairs for some time. It has a collar with a telephone number. You call on the phone and what do you say?

(*Original version in English: A cat has strolled into your yard. It has a collar with a telephone number. You call and say… Reason for modification: The majority of people in Hong Kong reside in buildings and do not have a yard.)
