# Supplementary material for: Validation of a Cantonese Version of the Amsterdam-Nijmegen Everyday Language Test (CANELT): A functional approach
Source: PLoS One. 2024 May 24;19(5):e0303810. doi: 10.1371/journal.pone.0303810 (PMC11125462; doi:10.1371/journal.pone.0303810)
Supplement: S2 Appendix — (DOCX) [file pone.0303810.s002.docx]

**Appendix B**

**The Scoring Criteria of the CANELT with Scoring Examples**

**Definition of the two measures**

Opening (O): Utterance(s) that illustrate the information provided in the scenario descriptions

New information (NI): Utterance(s) that provide additional information not explicitly stated in the situation descriptions.

**Scoring Criteria**

The maximum scores in Opening and New Information are 20, respectively. The response will be scored in each question with either 0, 0.5, or 1 credit. An utterance will be considered complete if it includes both subject/object and predicate. 0 credit will be scored if no response or irrelevant verbal responses are given.

- 1 credit: Production of at least one piece of complete content related to O or NI of the scenario
- 0.5 credit: Production of accurate but incomplete content related to O or NI of the scenario
- 0 credit: No response / irrelevant response

**Scoring Examples**

Alternative answers that fulfill the scoring criteria are acceptable.

Question 3: 我哋喺間鋪頭入面。你想買電視，我係個店員，我同你講:「有咩可以幫到你呀?」你跟住會講啲咩?

(We are in a shop, and you want to buy a television. I am the salesperson and I ask, “Can I help you?” What do you say?)

Examples of Opening with:

- 1 credit: 買電視 (**buy** a **television**.)／我想換電視 ( I want to **change/replace** to a new **television**.)
- 0.5 credit: 買 (buy)／電視 (television)
- 0 credit: nil / other unrelated information

Examples of New Information:

- 1 credit: 可唔可以介紹吓？(Do you **have** any **recommendations**?／我睇下先 (I am just **looking around**)／有冇呢個牌子？(Do you **have** this **brand**?)
- 0.5 credit: 清啲 (Clearer)／牌子 (brand)
- 0 credit: nil / other unrelated information

Question 4: 你拎咗一對鞋去鞋鋪整。對鞋有幾度爛咗，但係你淨係想整個鞋頭。你會點同個店員講呢?

(You are taking a pair of shoes to the shoe repairs. The shoes need to be repaired in several places, but for some reason you want to repair the toe cap only. What do you say?)

Examples of Opening with:

- 1 credit: 幫我**整**個**鞋頭** (help me **repair** the **toe cap**.)／我對鞋有幾度爛咗，但係我淨係想整個鞋頭。 (The shoes need to be repaired in several places, but I just want to **repair** the **toe cap**.)
- 0.5 credit: 整(repair/fix) /鞋頭(toe cap)／對鞋(the shoes)／爛 (broken)
- 0 credit: nil / other unrelated information

Examples of New Information:

- 1 credit: 要幾多錢? (**How much** is it?)／要等幾耐?(**How long** does it take?)
- 0.5 credit: 平(cheaper) / 錢 (money)
- 0 credit: nil/ other unrelated information
