# Supplementary material for: Validation of a Cantonese Version of the Amsterdam-Nijmegen Everyday Language Test (CANELT): A functional approach
Source: PLoS One. 2024 May 24;19(5):e0303810. doi: 10.1371/journal.pone.0303810 (PMC11125462; doi:10.1371/journal.pone.0303810)
Supplement: S3 Appendix — (DOCX) [file pone.0303810.s003.docx]

**Appendix C**

**Post-hoc Comparisons of Healthy Participants Across Different Age Groups**

| Games-Howell Post-Hoc Test – Opening (raw scores) | | | | | | | | | | | |
| --- | --- | --- | --- | --- | --- | --- | --- | --- | --- | --- | --- |
|  |  | **1** |  | **2** |  | **3** |  | **4** |  |  |  |
| **1** (30-39 years) | Mean difference | — |  | 0.15 |  | 0.325 |  | 1.98 | ** |  |  |
|  | t-value | — |  | 0.38 |  | 0.926 |  | 3.83 |  |  |  |
|  | df | — |  | 31.7 |  | 35.0 |  | 26.2 |  |  |  |
|  | p-value | — |  | 0.995 |  | 0.885 |  | 0.006 |  |  |  |
| **2** (40-49 years) | Mean difference |  |  | — |  | 0.175 |  | 1.82 | * |  |  |
|  | t-value |  |  | — |  | 0.398 |  | 3.15 |  |  |  |
|  | df |  |  | — |  | 36.9 |  | 34.4 |  |  |  |
|  | p-value |  |  | — |  | 0.994 |  | 0.026 |  |  |  |
| **3** (50-59 years) | Mean difference |  |  |  |  | — |  | 1.65 | * |  |  |
|  | t-value |  |  |  |  | — |  | 3.00 |  |  |  |
|  | df |  |  |  |  | — |  | 31.1 |  |  |  |
|  | p-value |  |  |  |  | — |  | 0.039 |  |  |  |
| **4** (60-69 years) | Mean difference |  |  |  |  |  |  | — |  |  |  |
|  | t-value |  |  |  |  |  |  | — |  |  |  |
|  | df |  |  |  |  |  |  | — |  |  |  |
|  | p-value |  |  |  |  |  |  | — |  |  |  |
| **5** (70-79 years) | Mean difference | 2.300 | ** | 2.150 |  | 1.975 |  | 0.325 |  |  |  |
|  | t-value | 4.418 |  | 3.681 |  | 3.564 |  | 0.485 |  |  |  |
|  | df | 26.0 |  | 34.2 |  | 30.8 |  | 38.0 |  |  |  |
|  | p-value | 0.001 |  | 0.007 |  | 0.010 |  | 0.988 |  |  |  |
| Games-Howell Post-Hoc Test – New Information (raw scores) | | | | | | | | | | | |
|  |  | **1** |  | **2** |  | **3** |  | **4** |  |  |  |
| 1 (30-39 years) | Mean difference | — |  | 0.350 |  | 1.100 |  | 2.65 | *** |  |  |
|  | t-value | — |  | 1.02 |  | 2.41 |  | 5.22 |  |  |  |
|  | df | — |  | 37.8 |  | 31.0 |  | 28.6 |  |  |  |
|  | p-value | — |  | 0.844 |  | 0.140 |  | < .001 |  |  |  |
| 2 (40-49 years) | Mean difference |  |  | — |  | 0.750 |  | 2.30 | *** |  |  |
|  | t-value |  |  | — |  | 1.61 |  | 4.47 |  |  |  |
|  | df |  |  | — |  | 32.2 |  | 29.7 |  |  |  |
|  | p-value |  |  | — |  | 0.501 |  | < .001 |  |  |  |
| 3 (50-59 years) | Mean difference |  |  |  |  | — |  | 1.55 |  |  |  |
|  | t-value |  |  |  |  | — |  | 2.60 |  |  |  |
|  | df |  |  |  |  | — |  | 37.3 |  |  |  |
|  | p-value |  |  |  |  | — |  | 0.092 |  |  |  |
| 4 (60-69 years) | Mean difference |  |  |  |  |  |  | — |  |  |  |
|  | t-value |  |  |  |  |  |  | — |  |  |  |
|  | df |  |  |  |  |  |  | — |  |  |  |
|  | p-value |  |  |  |  |  |  | — |  |  |  |
| 5 (70-79 years) | Mean difference | 3.400 | *** | 3.050 | *** | 2.300 | * | 0.750 |  |  |  |
|  | t-value | 5.50 |  | 4.89 |  | 3.32 |  | 1.03 |  |  |  |
|  | df | 25.2 |  | 26.0 |  | 33.6 |  | 36.0 |  |  |  |
|  | p-value | < .001 |  | < .001 |  | 0.017 |  | 0.839 |  |  |  |

*Note*. * p < .05, ** p < .01, *** p < .001
