# Supplementary material for: Validation of a Cantonese Version of the Amsterdam-Nijmegen Everyday Language Test (CANELT): A functional approach
Source: PLoS One. 2024 May 24;19(5):e0303810. doi: 10.1371/journal.pone.0303810 (PMC11125462; doi:10.1371/journal.pone.0303810)
Supplement: Attachment — Submitted filename: Responses to Reviewers.docx [file pone.0303810.s004.docx]

**Responses to Reviewers**

Dear Editor and reviewers,

Thanks very much for your comments. They are all taken seriously. Please find my responses to each of the comments below.

Thank you very much and I am looking forward to receiving your reply.

Best,

Winsy Wong

Journal Requirements:

When submitting your revision, we need you to address these additional requirements.

1. Please ensure that your manuscript meets PLOS ONE's style requirements, including those for file naming. The PLOS ONE style templates can be found at
   <https://journals.plos.org/plosone/s/file?id=wjVg/PLOSOne_formatting_sample_main_body.pdf> and 
   <https://journals.plos.org/plosone/s/file?id=ba62/PLOSOne_formatting_sample_title_authors_affiliations.pdf>

**Response**: The style requirements in the manuscript and file naming have been corrected in the resubmission.

2. Note from Emily Chenette, Editor in Chief of PLOS ONE, and Iain Hrynaszkiewicz, Director of Open Research Solutions at PLOS: Did you know that depositing data in a repository is associated with up to a 25% citation advantage (<https://doi.org/10.1371/journal.pone.0230416>)? If you’ve not already done so, consider depositing your raw data in a repository to ensure your work is read, appreciated and cited by the largest possible audience. You’ll also earn an Accessible Data icon on your published paper if you deposit your data in any participating repository (<https://plos.org/open-science/open-data/#accessible-data>).

**Response:** Thank you for the suggestion. The raw data has been uploaded to OSF (<https://osf.io/p3ehr/files/osfstorage/65bf0c659b32ca077097f691>). The availability of the raw data file is mentioned in the data availability statement (p. 29; lines 501-502).

3. Please review your reference list to ensure that it is complete and correct. If you have cited papers that have been retracted, please include the rationale for doing so in the manuscript text, or remove these references and replace them with relevant current references. Any changes to the reference list should be mentioned in the rebuttal letter that accompanies your revised manuscript. If you need to cite a retracted article, indicate the article’s retracted status in the References list and also include a citation and full reference for the retraction notice.

**Response:** The reference list has been reviewed again. To our understanding no retracted article is cited in the manuscript.


[Note: HTML markup is below. Please do not edit.]

Reviewers' comments:

Reviewer's Responses to Questions

**Comments to the Author**

1. Is the manuscript technically sound, and do the data support the conclusions?

The manuscript must describe a technically sound piece of scientific research with data that supports the conclusions. Experiments must have been conducted rigorously, with appropriate controls, replication, and sample sizes. The conclusions must be drawn appropriately based on the data presented.

Reviewer #1: Partly

Reviewer #2: Yes

2. Has the statistical analysis been performed appropriately and rigorously?

Reviewer #1: Yes

Reviewer #2: Yes

3. Have the authors made all data underlying the findings in their manuscript fully available?

The [PLOS Data policy](http://www.plosone.org/static/policies.action#sharing) requires authors to make all data underlying the findings described in their manuscript fully available without restriction, with rare exception (please refer to the Data Availability Statement in the manuscript PDF file). The data should be provided as part of the manuscript or its supporting information, or deposited to a public repository. For example, in addition to summary statistics, the data points behind means, medians and variance measures should be available. If there are restrictions on publicly sharing data—e.g. participant privacy or use of data from a third party—those must be specified.

Reviewer #1: Yes

Reviewer #2: Yes

4. Is the manuscript presented in an intelligible fashion and written in standard English?

PLOS ONE does not copyedit accepted manuscripts, so the language in submitted articles must be clear, correct, and unambiguous. Any typographical or grammatical errors should be corrected at revision, so please note any specific errors here.

Reviewer #1: Yes

Reviewer #2: Yes

5. Review Comments to the Author

Please use the space provided to explain your answers to the questions above. You may also include additional comments for the author, including concerns about dual publication, research ethics, or publication ethics. (Please upload your review as an attachment if it exceeds 20,000 characters)

Reviewer #1: The study validated the Cantonese version of the Amsterdam-Nijmegen Everyday Language Test (CANELT) for individuals with aphasia. Using quantitative scoring, it assessed communication abilities in both aphasic and healthy individuals. Responses from 56 people with aphasia and 100 healthy Cantonese speakers were orthographically transcribed and analyzed for completeness and information. The author provides a detailed description of the task, its real-life application, and compares it with other available assessments like ASHA FACS.

**Comment:** The differences between ANELT and CANELT lack clarity, and the rationale for using CANELT over ANELT needs further explanation.
**Response:** The rationale for using CANELT over ANELT is explained on page 7 (lines 116-118). And the differences between ANELT and CANELT are included in p. 10 (lines 164-169).

**Comment:** The present study demonstrates the adaptation of CANELT for Cantonese speakers, combining data from current and previous studies. However, it's unclear if the same protocol was consistently applied across these studies for test administration and results analysis. Clarity is needed regarding the re-analysis of orthographic transcriptions from previous studies and any considerations for inter-rater consistency in transcription in both present and previous studies.
**Response:** The same protocol was consistently applied across studies for test administration (p. 13, lines 225-226) and data analysis (p 15, lines 250-253). To further ensure inter-rater consistency across studies, inter-rater reliability was established based on scores obtained from the two previous studies, i.e., Yip (2019) and Lor (2020). Satisfactory results are obtained and updated in the abstract (p. 3, line 43) and main text (p. 23, line 395).

**Comment:** Further information is necessary regarding participant recruitment and diagnosis. Were assessments conducted by registered speech pathologists, psychologists, or neurologists? How were neurodegenerative conditions ruled out?
**Response:** Assessments were conducted by the author (who is a qualified speech therapist in Hong Kong or by trained speech therapy students under her supervision (p. 11, lines 185-187). Neurodegenerative conditions were based on the medical reports/ reports given by the PWA/caretakers. Such information has been added in the Method Section (p.10-11, lines 181-184)

**Comment:** Concerning Table 1, while the left column presents data from healthy adults and the right from people with aphasia, the final row seems to reference fluent/non-fluent aphasia in PWA, with specific aphasia types listed on the right. To avoid confusion, the table format could be split into two or restructured for clarity, as the current presentation may be misleading.
**Response:** Table 1 has been restructured to report the aphasia subtypes of PWA (p.12).

The results' presentation justifies the statistical analysis conducted.

Detailed procedures for translation and cultural adaptation from Dutch to Cantonese are provided.

Overall, the study significantly contributes to cross-linguistic research and the expansion of culturally adapted, language-specific assessment tools

Reviewer #2: In paper Validation of a Cantonese Version of the Amsterdam Nijmegen Everyday Language Test (CANELT): A functional approach autor test the psychometric properties of the ANELT in Cantonese. The paper is well written and the way the data is analysed is very interesting, i.e. it differs significantly from similar studies. Namely, the author provides an overview of how the Cantonese authors develop the scoring system for the CANELT. Analyses based on main concepts provide direct information about the informativeness of discourse production at the macrostructure level as well as opening and new information at the propositional level. In this way, the authors have developed a comprehensive approach to analysing discourse processing and production, which is a prerequisite for effective functional communication. I can recommend the publication of the paper. I have only a few comments that should be taken into account:
**Comment:** Line 61: Can you conclude the sentence with an example of some of the tools; among them, please mention the Scenario Test, as you mention it later in the manuscript. The same sentence – several assessment tools for measuring discourse production…. – actually, there should be - several assessment instruments to measure functional communication based on monologic discourse… because there are many instruments that measure discourse production but not functional communication. For example, in the Boston test or CAT test, there are picture description tasks that measure discourse production but not functional communication.

**Response:** Thank you for the suggestion. Some other functional communication measures, including the Scenario Test, have been included and mentioned (p. 4, lines 62-67).

**Comment:** Line 81 – can you very briefly define Comprehensibility A-Scale and Intelligibilty B-Scale

**Response:** The definitions of both scales are included (p.5; lines 84-86).

**Comment:** You use different terms for the control group – neurological healthy individuals, normal controls, healthy normals - ... - please use a single term and use it consistently throughout the manuscript.

**Response:** The term ‘neurologically healthy controls’ is used to refer to the control participants of the current study throughout the manuscript for consistency.

**Comment:** In the "Participants" section, provide additional demographic data of PwA – are they all monolingual? What is their level of education? What is their gender?

**Response:** The language use and level of education of PWA are included in p. 11; lines 188-190. Gender distribution is included in Table 1 (p.12)

**Comment:** Line 174 – Indicate the exact number of subjects taken from other authors: ...the rest were based on data collected in other studies: n=? from Law and Lo`s (15), n=? form Yip`s (18) and n=? from Lor`s study (19)

**Response:**
The exact number of subjects taken from each study is now included (p. 11, lines 194-196).

**Comment:** Line 185 – is the Duch-speaking aphasiologist the author of the ANELT? It is important to have the author's permission to translate the test into another language or the publisher's copyright – this is ethically more important than knowing who gave you the test.

**Response:** We tried to contact the author of the test but he passed away some time ago. We contacted the publisher and has obtained an agreement ‘*Translation from the ANELT/ANTAT, copyright 2008 Hogrefe Uitgevers BC (Amsterdam, the Netherlands) and Cognition Products BV – L. Blomert*’ for translating the test and dissemination of research findings (p. 12, lines 206-208) in journal articles. Under the current agreement, we are not allowed to publish, distribute or upload the content of CANELT. Therefore, Supplementary Material 1 (S1) has been modified only to include the five modified scenarios (in their English translated version) and their reasons for the modification.

**Comment:** Lines 191 and 192 – can you briefly describe five situations that were culturally inappropriate – this may be interesting for readers when confronted with a specific example of cultural bias.

**Response:** The five situations where modifications were made are specified in S1 Appendix.

**Comment:** Data analysis section – can you give a very brief and basic definition of each parameter of validity and reliability?

**Response**: A brief definition of each parameter of validity and reliability is given in the respective section (p. 16, lines 270-271, 277-279; p. 16-17, 284-290; p. 17, 297-299,p.18, 309-310, 314-323; p. 19, lines 325-329).

**Comment:** Line 254 – do not use "people with/without aphasia" – when I first read this sentence I thought you were talking about people who have had a stroke but have and do not have aphasia; in fact you are talking about PwA and healthy speakers – this is another example of why it is important that you use terminology consistently.

**Response:** The term ‘neurologically healthy controls’ has been used throughout the manuscript to avoid confusion.

**Comment:** Lines 266 – 267 – please revise the last part of the sentence - Items with low face validity, i.e., 30% or more respondents rated the item as ‘poor’ in assessing one’s communication ability, were excluded– should be excluded.
**Response:** Revision has been made accordingly (p.18, line 307).

**Comment**: Line 349 - which serves as a diagnostic tool – for what? Add: functional communication of Cantonese-speaking PwA

**Response:** Revision has been made in the respective content (p. 23, lines 401-402).
