# Supplementary material for: Validation of a Cantonese Version of the Amsterdam-Nijmegen Everyday Language Test (CANELT): A functional approach
Source: PLoS One. 2024 May 24;19(5):e0303810. doi: 10.1371/journal.pone.0303810 (PMC11125462; doi:10.1371/journal.pone.0303810)
Supplement: Attachment — Submitted filename: Responses to Reviewers.docx [file pone.0303810.s005.docx]

**Responses to Reviewers**

Dear Editor and reviewers,

Thanks very much for your comments. They are all taken seriously. Please find my responses to each of the comments below.

Thank you very much and I am looking forward to receiving your reply.

Best,

Winsy Wong

Journal Requirements:

Please review your reference list to ensure that it is complete and correct. If you have cited papers that have been retracted, please include the rationale for doing so in the manuscript text, or remove these references and replace them with relevant current references. Any changes to the reference list should be mentioned in the rebuttal letter that accompanies your revised manuscript. If you need to cite a retracted article, indicate the article’s retracted status in the References list and also include a citation and full reference for the retraction notice.

**Response:** The reference list has been reviewed.

[Note: HTML markup is below. Please do not edit.]

Reviewers' comments:

Reviewer's Responses to Questions

Comments to the Author

1. If the authors have adequately addressed your comments raised in a previous round of review and you feel that this manuscript is now acceptable for publication, you may indicate that here to bypass the “Comments to the Author” section, enter your conflict of interest statement in the “Confidential to Editor” section, and submit your "Accept" recommendation.

Reviewer #1: All comments have been addressed

Reviewer #2: All comments have been addressed

2. Is the manuscript technically sound, and do the data support the conclusions?

The manuscript must describe a technically sound piece of scientific research with data that supports the conclusions. Experiments must have been conducted rigorously, with appropriate controls, replication, and sample sizes. The conclusions must be drawn appropriately based on the data presented.

Reviewer #1: Yes

Reviewer #2: Yes

3. Has the statistical analysis been performed appropriately and rigorously?

Reviewer #1: Yes

Reviewer #2: Yes

4. Have the authors made all data underlying the findings in their manuscript fully available?

The PLOS Data policy requires authors to make all data underlying the findings described in their manuscript fully available without restriction, with rare exception (please refer to the Data Availability Statement in the manuscript PDF file). The data should be provided as part of the manuscript or its supporting information, or deposited to a public repository. For example, in addition to summary statistics, the data points behind means, medians and variance measures should be available. If there are restrictions on publicly sharing data—e.g. participant privacy or use of data from a third party—those must be specified.

Reviewer #1: Yes

Reviewer #2: Yes

5. Is the manuscript presented in an intelligible fashion and written in standard English?

PLOS ONE does not copyedit accepted manuscripts, so the language in submitted articles must be clear, correct, and unambiguous. Any typographical or grammatical errors should be corrected at revision, so please note any specific errors here.

Reviewer #1: Yes

Reviewer #2: Yes

6. Review Comments to the Author

Please use the space provided to explain your answers to the questions above. You may also include additional comments for the author, including concerns about dual publication, research ethics, or publication ethics. (Please upload your review as an attachment if it exceeds 20,000 characters)

Reviewer #1: Thank you for the opportunity to review the revised version of the manuscript. I acknowledge that all issues raised in the previous review round have been satisfactorily addressed.

Reviewer #2: As I said in my first review, I like the paper because it is not a classical, i.e. rather technical paper that only provides data on the validity and reliability of the test. This paper contains detailed information about the reasons (motivations) for the development of the CANELT, its advantages and disadvantages and, most importantly, about all the innovations that various authors have introduced over the last five years of its development to improve it in part of the test items and in the scoring section.

I have only a few small corrections:

Line 66: I would not say: among these measures …rather say Among these assessment tools…

**Response:** The suggested amendment has been made (p. 4, line 66).

In general, I would not speak of normative performance, since performance is always variable and therefore needs to be normed – I would replace this syntagm normative performance accordantly in the paper with normative data (e.g. lines 192, 261, 267, etc).

**Response:** The term ‘normative data’ has been used to replace ‘normative performance’ (lines 69, 192, 256-257, 261, 267).

The sentence in lines 68-70 has the same content as the sentence in lines 80-82. Delete the sentence in lines 80-82.

**Response:** The sentence in lines 80-82 has been deleted (p. 5, lines 80-82).

Line 86: explanation of B-Scale is somewhat unclear – I recommend that you add…examines the perception of utterances in such a way that words are recognizable regardless of their meaning.

**Response:** The suggested change has been made (p. 5, lines 86-87).

Line 158 – add reference number [24] after Lor

**Response:** Reference number [24] has been added after Lor (p. 9, line 158).

Line 186 – please use the terminology speech-language therapist and use this term accordingly (e.g., in line 223).

**Response:** The terminology ‘speech-language therapist’ has been used consistently in the manuscript (p. 11, line 186; p. 13, line 223-224).

Line 223 – add trained – who was either a trained speech-language therapy student…

**Response:** The word ‘trained’ has been added (p. 13, line 223).

Line 187 – delete therapy – The trained students had ….

**Response:** The word ‘therapy’ has been deleted (p. 11, line 187).

Line 272 – add neurologically – among neurologically healthy controls…

**Response:** The word ‘neurologically’ has been added (p. 16, line 272).

Lines 341 -351 – the results show a decrease in performance on the variables Opening and New Information in both groups – it would be interesting to hear the author’s explanation for these results, especially for neurologically healthy controls, in the discussion section. (*This is just an idea for a new study - it would be intersting to analyse the data from neurologically healthy controls in two age groups only – for example, younger adults (30 to 59 years) and older (60+ years), as the data presented in this paper shows a trend of performance change around age 60. This aligns with Lor`s idea in lines 158-161, who attempted to develop age-based norms – perhaps with such a wide range of age groups, age differences can be captured).

**Response:** The decrease in performance in CANELT and its possible relation with cognitive decline associated with aging has been included in the Discussion (p. 27-28, lines 472-496). However, the aging effect observed in the current study should be treated with caution, given its limitations (p. 27, lines 481-485). It is suggested that such a topic should deserve further investigation (p. 28, lines 492-496).
